# From Trauma to Functional Asplenia: A Rare Case of Delayed Splenic Liquefaction and Infection

**DOI:** 10.7759/cureus.107035

**Published:** 2026-04-14

**Authors:** Sai Roopali Susarla, Trinayana Sri Yeduru, Arjuna Gera, Venkata Hanuman Phani Kumar Koppu

**Affiliations:** 1 Surgery, Gayatri Vidya Parishad Institute of Healthcare and Medical Technology, Visakhapatnam, IND; 2 General Surgery, Gayatri Vidya Parishad Institute of Healthcare and Medical Technology, Visakhapatnam, IND

**Keywords:** blunt abdominal trauma, functional asplenia, liquefactive transformation, nephrotic syndrome, splenic abscess mimic, splenic injury, streptococcal pneumonia

## Abstract

Delayed complications following blunt splenic trauma are uncommon and typically present within weeks to months. Late presentations occurring years after injury are rare and may pose diagnostic challenges, particularly when imaging mimics infectious pathology.

We report a case of a 28-year-old male with features suggestive of nephrotic syndrome, who presented with abdominal pain, edema, and constitutional symptoms. Imaging performed six months prior had suggested a splenic abscess; however, further evaluation revealed diffuse hypoattenuation of the spleen with absence of contrast enhancement, consistent with complete splenic devascularization. The patient had a remote history of blunt abdominal trauma two years prior, which had remained untreated. Ultrasound-guided aspiration yielded sterile, necrotic fluid, favoring a chronic post-traumatic process rather than active infection. Microbiological evaluation revealed streptococcal pneumonia. The findings were consistent with chronic splenic injury with near-complete liquefactive transformation leading to functional asplenia in the background of nephrotic syndrome. The patient was managed conservatively with antibiotics and supportive care.

This case highlights an unusual delayed presentation of severe splenic injury occurring years after trauma, resulting in functional asplenia and increased susceptibility to infection. It underscores the importance of correlating imaging findings with remote trauma history and considering functional asplenia in patients with extensive splenic devascularization. Early recognition of such atypical presentations is essential to guide appropriate management and prevent complications.

## Introduction

The spleen is the most frequently injured intra-abdominal organ in blunt abdominal trauma, with injuries ranging from minor capsular tears to complete vascular disruption [1]. The management of splenic trauma has evolved from routine surgical intervention to selective non-operative management in hemodynamically stable patients, leading to improved splenic preservation and increased recognition of delayed complications [2].

These complications typically occur within weeks to months following injury and include splenic abscess, infarction, delayed hemorrhage, and pseudocyst formation [3,4]. The incidence of such complications remains relatively low but is increasingly reported due to advances in imaging modalities and widespread use of non-operative strategies. Despite improved detection, these sequelae are generally confined to the early post-traumatic period and are rarely observed beyond a few months after the initial insult [3-5].

Delayed presentations occurring years after trauma are exceedingly rare and remain poorly characterized in the literature. Such extensive splenic damage may result in functional asplenia, a state of impaired immune function associated with increased susceptibility to infections, particularly by encapsulated organisms [6]. We report a case of near-complete liquefactive transformation of the spleen identified two years after blunt abdominal trauma, associated with functional asplenia and subsequent streptococcal pneumonia in a patient with underlying nephrotic syndrome. The uniqueness of this case lies in the unusually prolonged latency, extensive splenic devascularization with near-total parenchymal replacement, and the development of infection in the setting of functional asplenia.

## Case presentation

A 28-year-old male presented to the surgical outpatient department with complaints of diffuse abdominal pain for one month, associated with intermittent constipation, exertional shortness of breath, and decreased urine output. He also reported progressive facial puffiness for the past four years.

His past medical history was notable for features suggestive of nephrotic syndrome, for which he had undergone a renal biopsy one year prior but did not follow up, and the report was unavailable. He had also been diagnosed with hypertension two years earlier and was prescribed telmisartan and diuretics, which he discontinued after 2-3 months. Notably, the patient sustained blunt abdominal trauma in a motor vehicle accident two years prior, for which he did not seek medical attention.

On examination, the patient was malnourished, afebrile, with a blood pressure of 130/90 mmHg and pulse rate of 106 beats/min. He had facial puffiness, scrotal edema, and bilateral pitting pedal edema up to the knees. Respiratory examination revealed reduced breath sounds on the right side, and abdominal examination showed distension with diffuse tenderness.

Laboratory investigations revealed anemia (hemoglobin 9.8 g/dL; Hb normal range in men: 13.5-17.5 g/dL), leukocytosis (12,600/mm³; normal leukocyte count 4500-11000) with neutrophilic predominance (95%), and elevated inflammatory markers (CRP 45 mg/L; normal range: <10 mg/L). Liver function tests showed elevated transaminases and markedly raised alkaline phosphatase, along with hypoalbuminemia (2.0-2.3 g/dL; normal serum albumin: 3.5-5.5 g/dL). Serum direct bilirubin and serum total bilirubin were normal. Serum creatinine was borderline elevated, while blood urea levels were within normal limits. Although urine examination did not reveal active proteinuria at the time of admission, his clinical presentation of bilateral pitting edema and hypoalbuminemia was highly suspicious for an underlying renal disorder.

**Figure 1:**
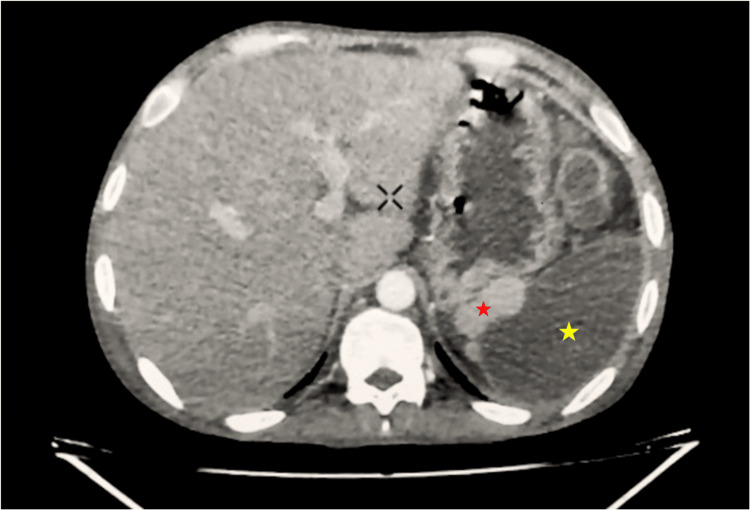
Contrast-enhanced CT abdomen (axial view) demonstrating markedly heterogeneous hypoattenuation of the splenic parenchyma with near-complete absence of contrast enhancement, consistent with extensive splenic devascularization The spleen is largely replaced by low-attenuation areas with internal heterogeneity, indicative of advanced liquefactive necrosis in the setting of chronic post-traumatic injury. The yellow star denotes regions of complete liquefaction (near fluid attenuation) within the spleen. The red star highlights adjacent hyperdense foci, likely representing residual vascular structures or contrast opacification near the splenic hilum.

Ultrasonography revealed a large, well-defined hypoechoic lesion occupying almost the entirety of the spleen, and a possibility of splenic abscess was considered. On further evaluation with contrast-enhanced computed tomography (Figure 1), the splenic parenchyma revealed diffuse hypoattenuation with non-enhancement on post-contrast images consistent with complete splenic devascularization. The splenic artery was not visualized and the splenic vein was attenuated. There were multiple well-defined round enhancing nodules noted in the perisplenic region, suggesting the possibility of accessory splenic tissue.

Microbiological evaluation revealed a sputum culture positive for Streptococcus species, sensitive to beta-lactam antibiotics and vancomycin but resistant to erythromycin. Blood cultures were sterile. Cytological analysis of aspirated splenic fluid showed predominantly neutrophils (95%) with necrotic debris, and cultures were negative.

The patient underwent ultrasound-guided aspiration of the splenic collection, yielding approximately 100 mL of thick, chocolate-colored fluid. He was managed conservatively with metronidazole 500 mg thrice a day for 10 days and cefoperazone+sulbactam 1.5 g twice a day for 10 days, along with diuretics, and supportive care. No surgical intervention was performed. During hospitalization, the patient showed symptomatic improvement and remained hemodynamically stable. He was discharged in stable condition.

## Discussion

This patient had features suggestive of underlying nephrotic syndrome and was non-compliant with treatment. A history of blunt abdominal trauma resulted in an untreated splenic injury, initially suspected as a splenic abscess, with findings later identified as near-complete liquefactive transformation of the spleen. This led to functional asplenia, predisposing the patient to the development of streptococcal pneumonia.

This case represents an unusual and delayed manifestation of severe splenic injury presenting as near-complete liquefaction and devascularization. The radiological findings, diffuse hypoattenuation, absence of contrast enhancement, non-visualization of the splenic artery, and attenuation of the splenic vein, are consistent with complete vascular compromise [7]. According to the American Association for the Surgery of Trauma (AAST) classification, these features correspond to a Grade V splenic injury, typically associated with acute presentation [7-9]. In contrast, our patient remained clinically stable and presented much later, highlighting an atypical disease course.

The most striking feature of this case is the prolonged latency between trauma and presentation. In existing literature, complications such as splenic abscess, infarction, or pseudocyst formation generally occur within weeks to a few months following injury [3]. Splenic abscesses are commonly reported within 4-6 weeks, and delayed rupture within two months [3,4]. Presentations beyond one year are rare, with only isolated reports describing delayed splenic abscesses or cystic transformations years after trauma [10-12]. Compared to these, the two-year interval observed in this case places it at the extreme end of the spectrum, suggesting a slow and clinically silent progression. Although rare, cases such as Toevs et al. reporting splenic abscess 10 years post-trauma demonstrate that delayed presentations, while uncommon, are possible [4].

The underlying mechanism likely involves an unrecognized splenic laceration with associated vascular injury, leading to progressive ischemia. Over time, infarcted splenic tissue undergoes liquefactive necrosis, forming a chronic fluid-filled cavity. While prior reports describe partial infarction or pseudocyst formation, most retain some degree of vascularity [12,13]. In contrast, our case demonstrated near-complete devascularization with extensive parenchymal replacement, representing a more advanced and rarely described endpoint of chronic splenic injury.

A key diagnostic challenge was distinguishing between a true splenic abscess and a chronic necrotic collection. Similar to previously reported cases, imaging findings initially suggested an abscess [4]. However, aspiration revealed sterile fluid with predominantly neutrophilic debris and no bacterial growth, favoring a non-infective etiology. This discrepancy underscores the importance of integrating radiological, microbiological, and clinical findings to avoid misdiagnosis and unnecessary treatment.

The presence of multiple splenunculi adds another dimension to this case. Accessory spleens are often considered to preserve some immune function in cases of splenic injury. However, despite their presence, the patient developed streptococcal pneumonia, suggesting inadequate immunological compensation. This finding aligns with the literature indicating that functional asplenia, even in the presence of accessory splenic tissue, may still confer significant susceptibility to infections, particularly with encapsulated organisms [6,14].

The presence of features suggestive of nephrotic syndrome may have further contributed to the patient’s vulnerability. Nephrotic syndrome is associated with immune dysfunction and increased infection risk. In previously reported cases, splenic abscesses are frequently linked to immunocompromised states such as diabetes or malignancy [15]. In this patient, the combination of nephrotic syndrome and functional asplenia likely had a synergistic effect, increasing susceptibility to infection.

From a management perspective, the patient was treated with ultrasound-guided aspiration and conservative measures, without requiring splenectomy. While splenectomy has traditionally been the standard treatment for splenic abscess, recent literature supports minimally invasive approaches in stable patients [16]. Our case supports this trend, demonstrating that conservative management can be effective even in complex presentations.

This case highlights several important clinical lessons. First, a history of remote trauma should be actively sought in patients presenting with atypical splenic lesions. Second, chronic post-traumatic splenic changes can closely mimic infectious processes, necessitating careful diagnostic correlation. Third, functional asplenia should be considered in cases of extensive splenic devascularization, regardless of the presence of accessory spleens. Finally, delayed presentations of splenic injury, though rare, should be recognized as a potential diagnostic entity, particularly in patients with underlying comorbidities.

## Conclusions

This case emphasizes a rare, delayed manifestation of severe splenic injury presenting years after trauma as near-complete liquefactive transformation and functional asplenia. It highlights the importance of thorough clinical history, careful radiologic-microbiologic correlation, and consideration of functional asplenia in atypical splenic lesions. Early recognition of such uncommon presentations is crucial to avoid misdiagnosis, guide appropriate management, and reduce the risk of infectious complications.
